# Expression of TP53 and IL-1α in unicystic ameloblastoma predicts the efficacy of marsupialization treatment

**DOI:** 10.1097/MD.0000000000009795

**Published:** 2018-02-09

**Authors:** Xinyu Zhang, Liu Liu, Xi Yang, Lizhen Wang, Chenping Zhang, Yongjie Hu

**Affiliations:** a Department of Oral-maxillofacial Head and Neck Oncology, Shanghai Ninth People's Hospital, College of Stomatology, Shanghai Jiao Tong University School of Medicine, Shanghai Key Laboratory of Stomatology; b Department of Oral Pathology, Shanghai Ninth People's Hospital, College of Stomatology, Shanghai Jiao Tong University School of Medicine, Shanghai, China.

**Keywords:** clinicopathology, IL-1α, marsupialization, TP53, unicystic ameloblastoma

## Abstract

In this study, we evaluated the effects of marsupialization in treating unicystic ameloblastoma (UA) and investigated the relationship between TP53 and interleukin 1 α (IL-1α) expression and the clinical outcome of UA treated with marsupialization.

Consecutive patients treated with marsupialization and curettage at Shanghai Ninth People's Hospital were included. According to the unified standard, 48 patients were included in this study. Of these, 20 showed a good response, 10 a partial response, and 18 no response, based on the outcome of the marsupialization procedure. The expression of proteins TP53 and IL-1α was detected with immunohistochemistry (IHC). The clinical and pathological characteristics of the patients were analyzed.

Analysis of the clinical and pathological characteristics showed that the effects of marsupialization treatment were significantly associated with lesion location (*P* < .001) and tumor diameter (*P* = .01). IHC showed that TP53 expression was significantly higher in the good-response group than in the partial- or no-response group (*P* = .02), and IL-1α expression was significantly higher in the good-response group than in the partial- and no-response groups (*P* = .03).

Marsupialization is an effective preliminary procedure for treating UA before curettage and peripheral ostectomy. The expression of the TP53 and IL-1α proteins correlates directly with the outcome of UA treated with marsupialization.

## 1 Introduction

Ameloblastoma is a slowly growing, locally aggressive epithelial odontogenic tumor of the jaw, with a propensity to recur but virtually no tendency to metastasize.^[1]^ According to the classification of tumors by the World Health Organization, the variants of ameloblastomas can be designated ameloblastoma, unicystic ameloblastoma (UA), extraosseous/peripheral ameloblastoma, and metastasizing (malignant) ameloblastom.^[1]^ Among the intraosseous ameloblastomas, 5% to 22% are the clinicopathological subtype known as UA.^[2]^

UA is an ameloblastoma variant that presents as a cyst with clinical and radiological characteristics of an odontogenic cyst, typically unilocular radiographic appearance, less aggressive behavior, and better response to conservative treatment. A histological examination shows a typical ameloblastomatous epithelium lining part of the cyst cavity, with or without luminal and/or mural tumor proliferation.^[2,3]^

The management of ameloblastoma is controversial because the biological behavior of this disease is unique. It is a slowly growing, locally invasive tumor with a high rate of recurrence. The management currently recommended for UA includes curettage with peripheral ostectomy. However, this can lead to subsequent bone defects. Marsupialization, a form of decompression, is another method for treating UA. The aim of marsupialization is to reduce the size of the UA so that less extensive surgery is required.^[4]^ Although many researchers have used marsupialization and curettage to treat UA, the inconsistent effectiveness of the technique remains its greatest drawback.

The TP53 protein is the product of the tumor suppressor gene *TP53*, and functions in G1 arrest to allow the repair of DNA damage and to prevent the cell from entering the S phase of the cell cycle, or alternatively to guide the damaged cell to apoptosis.^[5]^ The expression of TP53 is closely related to the prognosis of many kinds of tumors, including breast, gastric, and lung cancers.^[6–8]^ Interleukin-1 (IL-1) is a major upstream proinflammatory (“alarm”) cytokine that affects immunity and hematopoiesis by inducing cytokine cascades.^[9]^ As reviewed by Lewis et al,^[10]^ IL-1 expression is elevated in human breast, colon, lung, head, and neck cancers and melanomas, and patients with IL-1-positive tumors generally have poor prognoses.

In this study, we evaluated the clinical effects of marsupialization in the treatment of UA, and also investigated the relationship between TP53 and IL-1α expression and prognosis after treatment with marsupialization.

## 2 Methods

This retrospective study was performed between January 2003 and June 2014, and was approved by the Ethics Committee of the Ninth People's Hospital, Shanghai Jiao Tong University School of Medicine (Shanghai, China). All the patients in the study were diagnosed with mandibular UA, which was confirmed with histological and imaging examinations. Each patient underwent a full course of treatment, including marsupialization and curettage with peripheral ostectomy, with complete imaging examinations before marsupialization, during the follow-up period, and after curettage. The period between the 2 operations (marsupialization and curettage with ostectomy) was determined by the degree of jaw-bone regeneration after marsupialization. The patients were followed-up every 3 months for at least 60 months after curettage, with strict radiographic follow-up.

During marsupialization, we removed a window of the cyst lining for histological analysis, with or without the removal of several teeth, and an obstructor was then made to preserve the opening until the curettage operation. Frequent irrigation was required to keep the cyst cavity clean. Patients were required to undergo clinical and radiographic examinations every 3 months to observe the degree of jaw-bone regeneration. Once the lesion was no longer shrinking, we curetted the lesion and performed peripheral ostectomy.

**Figure 1:**
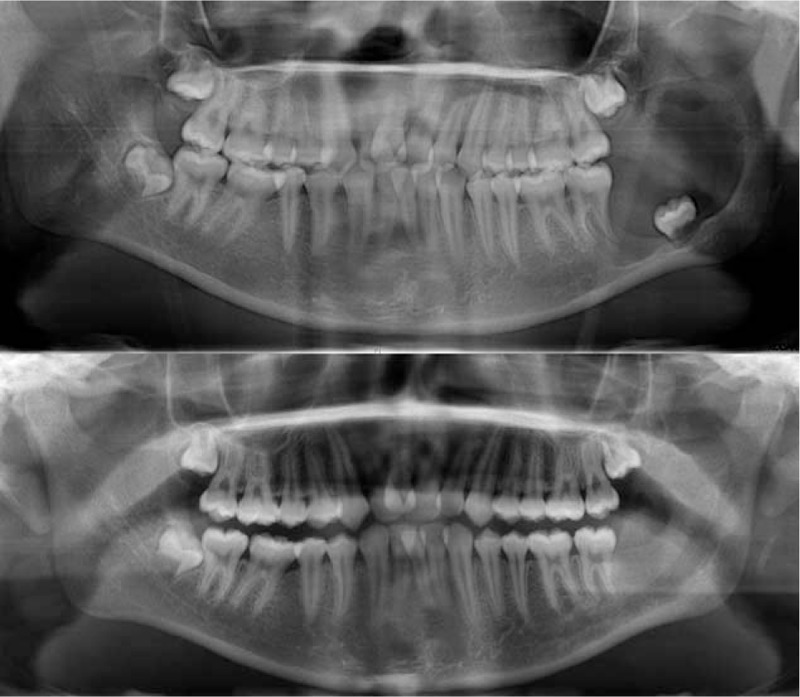
The radiographs (A) before marsupialization and (B) 8 months after marsupialization.

During curettage, we not only curetted the neoplastic lining, based on dental computerized tomography (CT), but also removed at least 0.5 cm of the mandible bone to obviate any potential tumor recurrence. We use CT scanning to evaluate the shrinkage (as the reduction in diameter), and the treatment response was evaluated based on the changes in the tumor diameter before and after marsupialization. The treatment responses were divided into 3 groups: good, partial, and none.^[11,12]^ A reduction in the maximum diameter of >50% was defined as good, of 26% to 50% was defined as partial, and of <25% was defined as none, as assessed with a CT scan (Fig. 1).

### 2.1 Immunohistochemistry (IHC)

A prediluted anti-TP53 antibody (clone DO-7; product code M7001; Glostrup, Dako, Denmark) and anti-IL-1α antibody (Abcam Anti-IL1 alpha antibody [AS5]; product code ab17281; Abcam, Cambridge, MA) were used for immunohistochemical staining. The tissues were first sequentially blocked with 3% hydrogen peroxide and normal serum. The tissue sections were incubated with the anti-TP53 antibody (1:200) or the anti-IL-1α antibody (1:50) at 4°C overnight and then with a biotinylated secondary antibody (EnVision Detection System Peroxidase/DAB+, Rabbit/Mouse; product code: K5007; Dako). Freshly prepared 3,3′-diaminobenzidine (DAB) was used to visualize the antigen–antibody reaction and hematoxylin was used for counterstaining. Oral squamous cell carcinoma was used as the positive control for TP53 and IL-1α staining, and PBS was used instead of the primary antibody solution in the negative control.

### 2.2 Evaluation of immunohistochemical staining

The cells were considered positive for the TP53 and IL-1α antigens if they displayed intranuclear DAB staining. The TP53 and IL-1α histological scores (H) were calculated according to Fromowitz as: H = P + i, where i is the intensity of staining (from 0 for negative cells to 3 for highly stained cells), and P is the percentage of positive cells, where *P* values of 0, 1, 2, 3, and 4 indicate 0% to 5%, 6% to 25%, 26% to 50%, 51% to 75%, and >75% positively stained cells, respectively. A minimum of 1000 cells were counted in each section. Tissue sections were scanned at 100× magnification for the most heavily labeled TP53- and IL-1α-positive cells in the epithelial linings. The cells were counted in 10 randomly selected fields on images at 400× magnification with conventional light microscopy, by 2 observers. Histological scores of 0–3 were considered negative, and scores of 4–7 were considered positive.

### 2.3 Statistical analysis

Data concerning the demographic, therapeutic, and experimental information were analyzed statistically with SPSS 16.0 for Windows, with a *χ*^2^ test, independent-samples nonparametric test, and Fisher exact test. The level of statistical significance was set at *P* < .05.

## 3 Results

The clinical characteristics of the patients are shown in Table 1. The study population consisted of 27 females and 21 males. The majority (66.67%) of patients were >18 years old. The lesion locations were: anterior region (6/12.50%), premolar region (8/16.67%), and molar–ramus (34/70.83%). The study included lesions with tumor diameters of 2 to 4 cm (13/27.08%), 4 to 6 cm (23/47.92%), and >6 cm (12/25.00%). Patients were treated with marsupialization for periods of 6 to 12 months (24/50.00%), 13 to 18 months (15/31.25%), or >18 months (9/18.75%). The average marsupialization time was 16.5 months (6–20.8 months). The percentage change in the radio density of UA ranged from −34.55% to 80.77%, with a mean reduction of 36.88%.

**Table 1:**
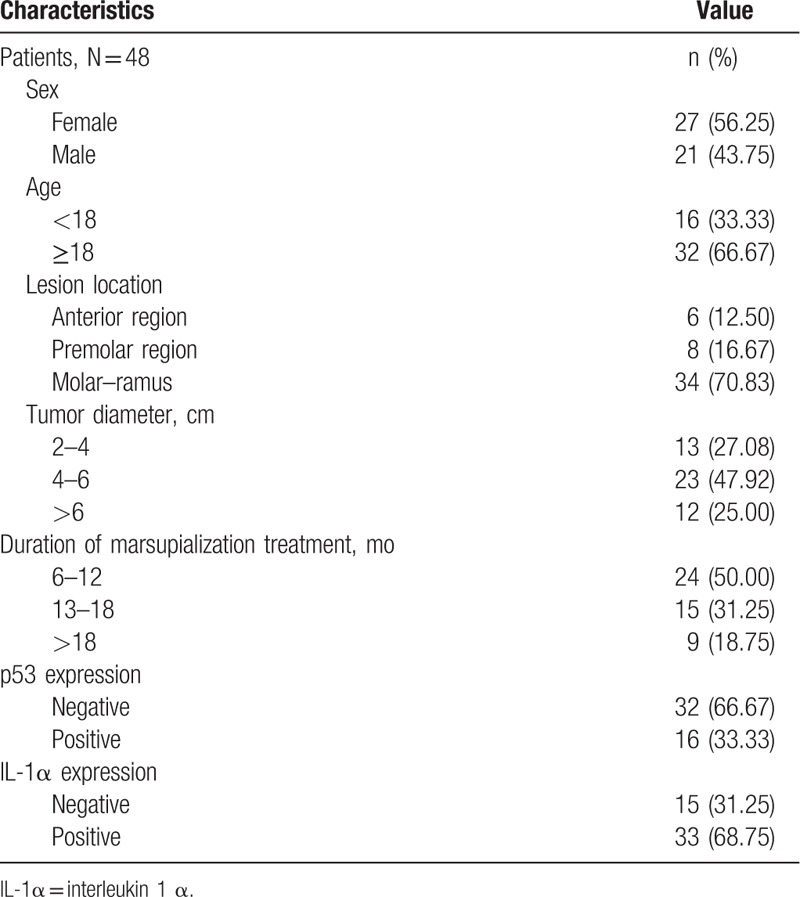
Patient and clinical characteristics.

To evaluate the roles of TP53 and IL-1α in the progression of marsupialization in UA, we immunohistochemically stained for TP53 and IL-1α in all tumors. TP53 expression was observed in 16 patients (33.33%), but was not observed in 32 patients (66.67%). IL-1α was expressed in 33 patients (68.75%), but not in 15 patients (31.25%).

### 2.4 Correlations between treatment response and clinicopathological parameters in marsupialized patients

The correlations between the treatment response and the clinicopathological features of the patients are summarized in Table 2. No significant correlations were observed between age and the duration of marsupialization. Marsupialization treatment was significantly associated with lesion location (*P* < .001), tumor diameter (*P* = .01), TP53 expression (*P* = .02), and IL-1α expression (*P* = .01). UA in molar–ramus had a better prognosis than UA in the anterior and premolar regions, and tumors <4 cm also had a good prognosis.

**Table 2:**
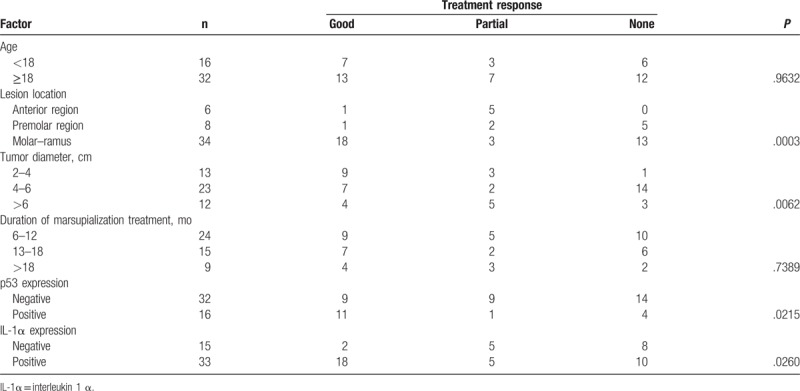
Clinicopathological features of the UA patients and their primary tumors and their association with p53 and IL-1α expression.

### 2.5 TP53 is overexpressed in UA and strongly associated with the efficacy of marsupialization

**Figure 2:**
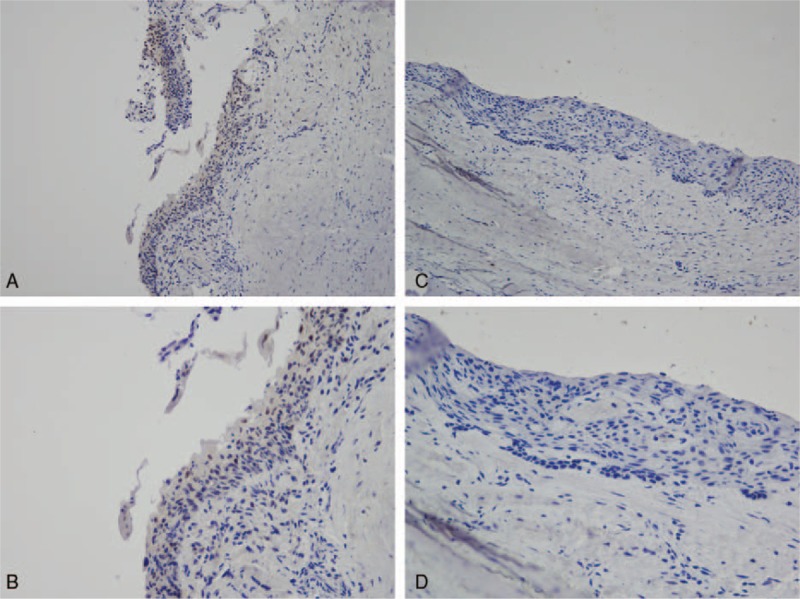
p53 expression in UA. (A) p53 expression in the effective group (200×). (B) p53 expression in the effective group (400×). (C) p53 expression in the ineffective group (200×). (D) p53 expression in the ineffective group (400×).

IHC revealed that TP53 protein was expressed in the basal and suprabasal cell layers of UA. We analyzed the relationship between TP53 and the clinicopathological features (including the therapeutic efficacy of marsupialization treatment) of the patients with UA. In the TP53-positive group, 68.75% of patients (11/16) showed a good response to marsupialization, 6.25% (1/16) showed a partial response, and 25.00% (4/16) did not respond to marsupialization. In contrast, of the patients in the TP-negative group, only 28.13% (9/32) had a good response, 28.13% (9/32) a partial response, and 43.75% (14/32) no response to marsupialization. These results demonstrated that TP53 expression was significantly higher in the good-response group than in the partial- or no-response group (*P* = .02) (Table 2, Fig. 2).

### 2.6 IL-1α is overexpressed in UA and strongly associated with the efficacy of marsupialization

**Figure 3:**
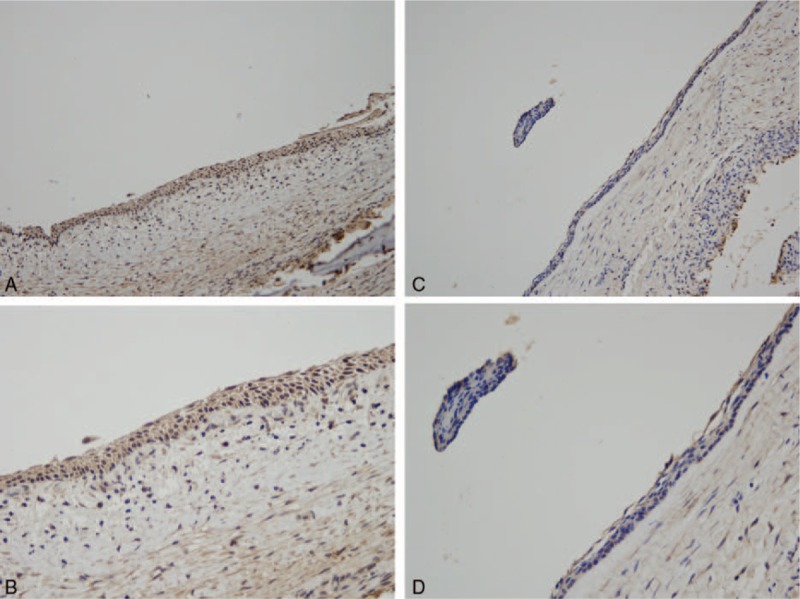
IL-1α expression in UA. (A) IL-1α expression in the effective group (200×). (B) IL-1α expression in the effective group (400×). (C) IL-1α expression in the ineffective group (200×). (D) IL-1α expression in the ineffective group (400×).

IHC revealed that the IL-1α protein was expressed locally in the epithelial cell cytoplasm in UA. The H score for IL-1α expression was based on staining intensity and the percentage of positive cells. We then analyzed the relationship between IL-1α and the treatment response to marsupialization in UA. In the IL-1α-positive group, 54.55% patients (18/33) showed a good response to marsupialization, 15.15% (5/33) a partial response, and 30.30% (10/33) no response. In the IL-1α-negative group, only 13.33% (2/15) showed a good response, 33.33% (5/15) a partial response, and 53.33% (8/15) no response to marsupialization. Thus, IL-1α expression was significantly higher in the good-response group than in the partial- or no-response group (*P* = .03; Table 2, Fig. 3).

The expression of both TP53 and IL-1α was elevated in the patients in whom the marsupialization procedure successfully minimized the tumor size, according to our criterion. The results indicated that the quantitative determination of TP53 and IL-1α predicted the outcome of marsupialization for UA.

During the follow-up period, none of the patients underwent malignant transformation. Throughout a follow-up period after curettage of at least 2 years, the recurrence rate of UA treated with marsupialization was 9.30% (4/43), and 5 patients were lost to follow-up. The recurrence rates were 5.56% (1/18) in the good-response group, 11.11% (1/9) in the partial-response group, and 12.50% (2/16) in the no-response group, which do not differ statistically significantly (*P* = .77).

## 4 Discussion

To the best of our knowledge, this is the largest study to evaluate the therapeutic response of UA to marsupialization and to investigate the relationship between TP53 and IL-1α expression and the clinical outcome of UA treated with marsupialization. Marsupialization can relieve the pressure within UA and allow new bone to fill in the defect, achieving partial success in reducing the UA size before further definitive surgery. In the present study, 41.67% (20/48) of the total patients had a good response to marsupialization, 20.83% (10/48) had a partial response, and 37.50% (18/48) had a poor response. The lesion location and tumor diameter were significantly associated with the therapeutic efficacy of marsupialization. Patients with higher TP53 expression showed a better response to marsupialization. IL-1α was overexpressed in UA and was significantly higher in the group displaying better therapeutic efficacy.

Although UA is a variant of ameloblastoma, it is considered to have a relatively benign biological behavior and a better response to conservative treatment, resulting in a better prognosis.^[2,3,13–15]^ UA is believed to be less aggressive than ameloblastomas and to respond more favorably to conservative management, including enucleation, curettage, and marsupialization.^[15,16]^ Marsupialization is reported to be a more effective treatment for cystic lesions than other treatments. Nakamura et al^[11]^ reported a series of 24 UAs treated with marsupialization, among which 16 lesions regressed to less than half their initial size. Because UA behaves less aggressively than other ameloblastomas and only recurs locally, we believe that marsupialization combined with curettage and peripheral ostectomy best maintains the continuity of the mandible and avoids mandibular-stage resection, ensuring a better quality of life for the patients. Even in the group that benefitted least, the interval between marsupialization and subsequent treatment procedures prolonged, due in part to the effects of the former treatment. According to Sampson and Pogrel,^[17]^ marsupialization is associated with a high recurrence rate because tumor cells may be left within the adjacent cancellous bone. However, the recurrence rate was low in our experiment. This may be because, based on our clinical experience, we curette with peripheral ostectomy to a depth of over 0.5 cm.

Why UA in the molar–ramus region benefited from marsupialization may be closely related to the anatomical structure and biomechanics of the mandible. The mandible is remodeled slowly through resorption under pressure and through bone regeneration under tension.^[18]^ The masticatory forces on the mandible mean that the molar–ramus receives and transmits most of the masticatory force, so the mandible undergoes the most active bone remodeling. The shape of the mandible is designed to withstand the masticatory force and transmits it to the base of the skull. Because of the mandibular anatomy, the molar–ramus bone density is greater than that in the anterior and premolar regions. This may lead to a better prognosis for UA treated with marsupialization, but further studies of this issue are required to test this proposition.

TP53 is a sequence-specific transcription factor that regulates the expression of many target genes. The biological functions of TP53 include the induction of cell-cycle arrest, apoptosis, or senescence in response to diverse stresses, thereby preventing cells from undergoing malignant transformation and tumorigenesis.^[19–22]^ The TP53 protein has a very short half-life and cannot be detected in normal tissues with immunohistochemical techniques, whereas a *TP53* gene mutation produces a protein with increased stability. The mutation of *TP53* is common in a wide range of human tumors and results in a more stable protein, allowing its detection with IHC. The level of TP53 expression in ameloblastoma is significantly higher than in the normal oral mucosa or in any other cystic lesion of the jaw.^[23]^ Previous research has shown that high TP53 expression indicates a poor prognosis in cancer patients, suggesting increased locally invasive behavior of the tumor and higher mitotic activity in epithelial cells. However, in our study, high TP53 expression predicted a better response to marsupialization in patients with UA.^[24]^ Why TP53 protein expression was elevated in the group of UA patients in whom marsupialization was effective can be explained by various factors peculiar to UA. TP53 regulates the expression of vascular endothelial growth factor (VEGF); higher VEGF expression stimulates angiogenesis, ultimately promoting osteogenesis in the mandibular bones, and the newly formed bone narrows the cyst.^[25]^ The overexpression of TP53 protein in UA may not be solely attributable to the mutation of the *TP53* gene, but to defects in protein degradation. Alternatively, the binding of TP53 to other proteins also stabilizes TP53 within the cell. Therefore, the presence of the wild-type TP53 protein in cells may explain the resulting good prognosis and sensitivity to marsupialization.^[23,26]^

IL-1 is a pleiotropic proinflammatory cytokine with biological activities that support its role in tumor growth.^[27]^ Several oncogenes, including RAS, MYC, and RET, not only mediate neoplastic transformation, but also activate intrinsic inflammatory cytokines that establish a proinvasive tumor microenvironment. Past research has shown that IL-1α plays an important role in bone resorption by inducing the production of matrix-metalloproteinase-like degradative enzymes and prostaglandins and/or the differentiation and activation of osteoclast-like cells.^[28]^ IL-1α may be closely related to the bone defect in UA. In this study, patients who expressed more IL-1α had a better response to marsupialization, perhaps because UA with high IL-1α expression shares the same biological behavior as odontogenic keratocysts (OKCs). In previous studies of OKC, high levels of IL-1α were found in the epithelial cells. Ninomiya et al^[29]^ reported that in OKC, marsupialization caused a rapid reduction in IL-1α expression and a decline in bone absorption. Therefore, the expression of IL-1α may be related to the process of bone resorption and may also indicate the presence of inflammation. The level of IL-1α expression can indicate whether the disease is in a progressive stage. Recent in vitro studies have shown that IL-1 stimulates epithelial cell proliferation directly and/or indirectly by inducing the secretion of several factors, such as keratinocyte growth factor, by interacting fibroblasts. The proliferation activity of epithelial cells is strongly related to the aggressiveness of cyst expansion in OKC.^[30]^ Previous research has demonstrated that IL-1α is strongly expressed in OKC, and that its expression decreases significantly after marsupialization.^[29]^ Many researchers have already confirmed that OKC responds well to marsupialization. Similarly, in our study, some patients with UA showed considerable sensitivity to marsupialization. Therefore, it is clear that the procedure is more effective in some patients than in others. However, the reason for this difference remains unclear. We hypothesize that the biological pattern of UA may cause small differences in the development of the disease. Further research is required to identify the cause of this phenomenon.

Extensive resection has been used to treat ameloblastomas to prevent possible recurrence, but it is associated with facial deformity and masticatory dysfunction.^[16]^ To optimize the therapeutic effect, a patient's facial esthetics, masticatory function, and quality of life must be considered. Many researchers have performed marsupialization before curettage to minimize the bone defect after surgery. However, because the effect of marsupialization varies from patient to patient, it is difficult to select the subsequent treatment when patients show a poor response in the marsupialization stage. In this study, we found that UA patients with high TP53 and IL-1α expression in the lesion responded better to marsupialization than those in whom the expression of these proteins was low, and therefore show a greater reduction of the cystic space after marsupialization.

Several of the findings of this study could contribute to the treatment of UA, improving the success rate of marsupialization and the evaluation of the prognosis of UA during the first surgery. IL-1α and TP53 levels were evaluated in pathological sections in this study. Although this procedure predicted the outcome of marsupialization, it required biopsies. A noninvasive and precise method of evaluating the expression of IL-1α and p53 is necessary to avoid overtreatment in the future.

In conclusion, our study shows that marsupialization combined with curettage and peripheral ostectomy is a good method for the treatment of UA because it maintains the continuity of the mandible, avoids or delays osteotomy, reduces the recurrence rate, and improves the patient's quality of life. Furthermore, UA associated with high TP53 and IL-1α expression predicts a better response to marsupialization than TP53- and IL-1α-negative UA.

## References

[1] Wright JM Vered M Update from the 4th Edition of the World Health Organization Classification of Head and Neck Tumours: odontogenic and maxillofacial bone tumors. Head Neck Pathol 2017;11:68–77. 28247226 10.1007/s12105-017-0794-1 PMC5340735

[2] Philipsen HP Reichart PA Unicystic ameloblastoma. A review of 193 cases from the literature. Oral Oncol 1998;34:317–25. 9861335 10.1016/s1368-8375(98)00012-8

[3] Ackermann GL Altini M Shear M The unicystic ameloblastoma: a clinicopathological study of 57 cases. J Oral Pathol 1988;17:541–6. 3150441 10.1111/j.1600-0714.1988.tb01331.x

[4] Lau SL Samman N Recurrence related to treatment modalities of unicystic ameloblastoma: a systematic review. Int J Oral Maxillofac Surg 2006;35:681–90. 16782308 10.1016/j.ijom.2006.02.016

[5] Kumar V Abbas AK Fausto N Robbins and Cotran Pathologic Basis of Disease. Philadelphia: Elsevier Saunders; 2005.

[6] Dookeran KA Dignam JJ Ferrer K p53 as a marker of prognosis in African-American women with breast cancer. Ann Surg Oncol 2010;17:1398–405. 20049641 10.1245/s10434-009-0889-3

[7] Kakeji Y Korenaga D Tsujitani S Gastric cancer with p53 overexpression has high potential for metastasising to lymph nodes. Br J Cancer 1993;67:589–93. 8439509 10.1038/bjc.1993.108 PMC1968266

[8] Quinlan DC Davidson AG Summers CL Accumulation of p53 protein correlates with a poor prognosis in human lung cancer. Cancer Res 1992;52:4828–31. 1324796

[9] Voronov E Dotan S Krelin Y Unique versus redundant functions of IL-1alpha and IL-1beta in the tumor microenvironment. Front Immunol 2013;4:177. 23847618 10.3389/fimmu.2013.00177 PMC3703603

[10] Lewis AM Varghese S Xu H Interleukin-1 and cancer progression: the emerging role of interleukin-1 receptor antagonist as a novel therapeutic agent in cancer treatment. J Transl Med 2006;4:48. 17096856 10.1186/1479-5876-4-48 PMC1660548

[11] Nakamura N Higuchi Y Tashiro H Marsupialization of cystic ameloblastoma: a clinical and histopathologic study of the growth characteristics before and after marsupialization. J Oral Maxillofac Surg 1995;53:748–54. discussion 746–755. 7595787 10.1016/0278-2391(95)90323-2

[12] Yong-Jie HU Si-Yi LI Li-Qun XU Clinical study of large mandibular odontogenic keratocyst treated by decompression. China J Oral Maxillofac Surg 2005;3:229–32.

[13] Gardner DG Corio RL Plexiform unicystic ameloblastoma. A variant of ameloblastoma with a low-recurrence rate after enucleation. Cancer 1984;53:1730–5. 6697311 10.1002/1097-0142(19840415)53:8<1730::aid-cncr2820530819>3.0.co;2-u

[14] Olaitan AA Adekeye EO Unicystic ameloblastoma of the mandible: a long-term follow-up. J Oral Maxillofac Surg 1997;55:345–8. discussion 349–350. 9120697 10.1016/s0278-2391(97)90122-1

[15] Pattipati S Ramaswamy P Praveen Kumar B Unicystic ameloblastoma. Int J Stomatol Occlusion Med 2013;6:33–7.

[16] Nakamura N Higuchi Y Mitsuyasu T Comparison of long-term results between different approaches to ameloblastoma. Oral Surg Oral Med Oral Pathol Oral Radiol Endod 2002;93:13–20. 11805772 10.1067/moe.2002.119517

[17] Sampson DE Pogrel MA Management of mandibular ameloblastoma: the clinical basis for a treatment algorithm. J Oral Maxillofac Surg 1999;57:1074–7. discussion 1078–1079. 10484108 10.1016/s0278-2391(99)90328-2

[18] Yuan X Luo S Shen G [Experimental study on selecting optimal time of orthodontic tooth movement into extraction sites]. Hua Xi Kou Qiang Yi Xue Za Zhi 2003;21:311–3. 14513593

[19] Kruse JP Gu W Modes of p53 regulation. Cell 2009;137:609–22. 19450511 10.1016/j.cell.2009.04.050 PMC3737742

[20] Levine AJ Oren M The first 30 years of p53: growing ever more complex. Nat Rev Cancer 2009;9:749–58. 19776744 10.1038/nrc2723 PMC2771725

[21] Vogelstein B Lane D Levine AJ Surfing the p53 network. Nature 2000;408:307–10. 11099028 10.1038/35042675

[22] Vousden KH Prives C Blinded by the light: the growing complexity of p53. Cell 2009;137:413–31. 19410540 10.1016/j.cell.2009.04.037

[23] Gadbail AR Patil R Chaudhary M Co-expression of Ki-67 and p53 protein in ameloblastoma and keratocystic odontogenic tumor. Acta Odontol Scand 2012;70:529–35. 21780975 10.3109/00016357.2011.600714

[24] de Vicente JC Torre-Iturraspe A Gutierrez AM Immunohistochemical comparative study of the odontogenic keratocysts and other odontogenic lesions. Med Oral Patol Oral Cir Bucal 2010;15:e709–15. 20383104 10.4317/medoral.15.e709

[25] Mukhopadhyay D Tsiokas L Sukhatme VP Wild-type p53 and v-Src exert opposing influences on human vascular endothelial growth factor gene expression. Cancer Res 1995;55:6161–5. 8521408

[26] Gadbail AR Chaudhary M Patil S Actual Proliferating Index and p53 protein expression as prognostic marker in odontogenic cysts. Oral Dis 2009;15:490–8. 19563416 10.1111/j.1601-0825.2009.01590.x

[27] Leon X Bothe C Garcia J Expression of IL-1alpha correlates with distant metastasis in patients with head and neck squamous cell carcinoma. Oncotarget 2015;6:37398–409. 26460957 10.18632/oncotarget.6054 PMC4741937

[28] Strand V Kavanaugh AF The role of interleukin-1 in bone resorption in rheumatoid arthritis. Rheumatology (Oxford, England) 2004;43 (suppl 3):iii10–6. 10.1093/rheumatology/keh202 15150427

[29] Ninomiya T Kubota Y Koji T Marsupialization inhibits interleukin-1alpha expression and epithelial cell proliferation in odontogenic keratocysts. J Oral Pathol Med 2002;31:526–33. 12269991 10.1034/j.1600-0714.2002.00029.x

[30] Suyama Y Kubota Y Ninomiya T Immunohistochemical analysis of interleukin-1 alpha, its type I receptor and antagonist in keratocystic odontogenic tumors. J Oral Pathol Med 2008;37:560–4. 18624935 10.1111/j.1600-0714.2008.00667.x

